# Targeted DNA Methylation Editing Using an All-in-One System Establishes Paradoxical Activation of *EBF3*

**DOI:** 10.3390/cancers16050898

**Published:** 2024-02-23

**Authors:** Rakesh Banerjee, Priyadarshana Ajithkumar, Nicholas Keestra, Jim Smith, Gregory Gimenez, Euan J. Rodger, Michael R. Eccles, Jisha Antony, Robert J. Weeks, Aniruddha Chatterjee

**Affiliations:** 1 Department of Pathology, Dunedin School of Medicine, University of Otago, Dunedin 9054, New Zealand; banra389@student.otago.ac.nz (R.B.); keeni175@student.otago.ac.nz (N.K.); jim.smith2@southerndhb.govt.nz (J.S.); gregory.gimenez@otago.ac.nz (G.G.); euan.rodger@otago.ac.nz (E.J.R.); michael.eccles@otago.ac.nz (M.R.E.); jisha.antony@otago.ac.nz (J.A.); 2 School of Health Sciences and Technology, UPES University, Dehradun 248007, India

**Keywords:** epigenomic editing, All-in-one CRISPR system, DNA methylation, paradoxical role, melanoma, RNA-sequencing

## Abstract

**Simple Summary:**

Contrary to the conventional understanding of DNA hypermethylation suppressing gene expression, a new mechanism emerges, suggesting that high methylation can paradoxically activate genes. This challenges the traditional notion that promoter methylation solely silences genes. The study employs a CRISPR-SunTag All-in-one system to manipulate the DNA methylation of the *EBF3* gene promoter segment in melanoma cells. Successful methylation and demethylation demonstrate the paradoxical role of DNA methylation, offering insights into the *EBF3* gene’s function, particularly in the IFN pathway signaling. This research challenges conventional views on methylation, providing potential insights into epigenetic drivers of malignancy and metastasis.

**Abstract:**

Cutaneous melanoma is rapidly on the rise globally, surpassing the growth rate of other cancers, with metastasis being the primary cause of death in melanoma patients. Consequently, understanding the mechanisms behind this metastatic process and exploring innovative treatments is of paramount importance. Recent research has shown promise in unravelling the role of epigenetic factors in melanoma progression to metastasis. While DNA hypermethylation at gene promoters typically suppresses gene expression, we have contributed to establishing the newly understood mechanism of paradoxical activation of genes via DNA methylation, where high methylation coincides with increased gene activity. This mechanism challenges the conventional paradigm that promoter methylation solely silences genes, suggesting that, for specific genes, it might actually activate them. Traditionally, altering DNA methylation in vitro has involved using global demethylating agents, which is insufficient for studying the mechanism and testing the direct consequence of gene methylation changes. To investigate promoter hypermethylation and its association with gene activation, we employed a novel approach utilising a CRISPR-SunTag All-in-one system. Here, we focused on editing the DNA methylation of a specific gene promoter segment (*EBF3*) in melanoma cells using the All-in-one system. Using bisulfite sequencing and qPCR with RNA-Seq, we successfully demonstrated highly effective methylation and demethylation of the *EBF3* promoter, with subsequent gene expression changes, to establish and validate the paradoxical role of DNA methylation. Further, our study provides novel insights into the function of the *EBF3* gene, which remains largely unknown. Overall, this study challenges the conventional view of methylation as solely a gene-silencing mechanism and demonstrates a potential function of *EBF3* in IFN pathway signalling, potentially uncovering new insights into epigenetic drivers of malignancy and metastasis.

## 1. Introduction

Among the extensively studied epigenetic modifications, DNA methylation is considered the most somatically heritable and stable mark [1,2]. The significance of DNA methylation in governing gene expression through epigenetic mechanisms is well-documented. Nevertheless, methylation also plays a pivotal role in numerous other scenarios, including X-chromosome inactivation, maintaining genomic stability, and regulating genomic imprinting [3,4]. Across this extensive array of functions, DNA methylation imparts an additional level of plasticity and dynamicity to genetic regulation, while still allowing for the stable transmission of a specific epigenotype during the process of cell replication. Given that the fundamental DNA sequence remains nearly uniform in all mammalian cells, the epigenetic profile is critical in moulding distinct cell lineages, making a substantial contribution to the diverse phenotypes observed [5]. Disruptions in the regulation of DNA methylation are associated with the pathogenesis of several diseases, especially cancer. Changes in DNA methylation, such as genome-wide hypomethylation and promoter hypermethylation of tumour-suppressor genes (TSGs), contribute to tumour development and progression [6,7,8]. This scope of global hypomethylation and promoter hypermethylation intensifies as the tumour progresses to metastasis, further amplifying the bimodal characteristics of the tumour DNA methylome [9]. Growing evidence from recent studies suggests a further defined role for site-specific DNA methylation changes acting as “epigenetic drivers” and potential markers in different cancer types that enable cancer growth [10,11,12,13].

Transcriptional regulation of a specific gene is influenced by DNA methylation, which modulates the recruitment of methyl CpG binding domain (MBD) proteins and transcriptional repressors. Conventionally, the lack of DNA methylation in gene promoter regions rich in CpG sites is linked to an ideal chromatin organisation that promotes active gene transcription. DNA hypermethylation of TSGs such as *CDKN2A* and *CDKN2B* has been demonstrated to result in their transcriptional silencing across various cancers [14]. A high level of methylation results in a condensed heterochromatin structure, leading to the subsequent inhibition of transcription [2,15,16]. The silencing of TSGs by hypermethylation contributes to cancer initiation, progression, and metastasis [17]. Conversely, global hypomethylation is recognised for its role in promoting gene expression changes, providing cancer cells with a selective advantage. This phenomenon affects genomic domains and contributes to genomic instability in cancer cells [18]. These fundamental concepts form the foundation of our current comprehension of how DNA methylation relates to gene expression. This mechanism of transcriptional silencing has been extensively investigated and is supported by a significant body of evidence accumulated over recent decades. While substantial evidence supports the classical paradigm, we and others have now established that promoter DNA methylation could be a mechanism of the paradoxical activation of genes in different contexts, such as metastasis and development [10,19,20]. In these scenarios, instances of active transcription from hypermethylated gene promoters have been documented, indicating an alternative role for DNA methylation as a facilitator of transcription. This paradoxical activity may be caused due to the binding of repressive transcription factors, distal element interactions, or alternative promoter expression. These possible molecular mechanisms of gene activation have been discussed in detail in our previous work [19].

Although not extensively studied, *EBF3* is known to play roles in the differentiation and migration of various cell types. *EBF3* is primarily known for its critical role in B-cell development; it acts as a master regulator in the commitment of progenitor cells to the B-cell lineage [21]. Dysregulation of *EBF3* expression can have significant consequences, leading to developmental abnormalities or disease conditions, particularly in cases where EBF3 acts as a master regulator, such as in B-cell development and neuronal differentiation. Moreover, it has been detected as a potential tumour suppressor in brain, breast, colorectal, gastric, liver, bone tumours, and acute myeloid leukaemia [10,21,22]. *EBF3* is often classically shown to be downregulated or mutated in certain malignancies, suggesting a potential tumour suppressor role [21]. Contrary to earlier findings, we identified *EBF3* as a putative epigenetic driver of metastasis in melanoma [10] and other cancer types [23], displaying the phenomenon of hypermethylation causing gene activation. Thus, *EBF3* in the context of melanoma presents a favourable framework to establish the mechanism of paradoxical gene expression. Hence, the work described in this study focuses on investigating *EBF3* as an example.

Despite the remarkable discoveries made in the field of DNA methylation and gene expression, it is important to note their limitations regarding the lack of validation of direct causality between promoter DNA methylation and ensuing gene expression. To advance and evaluate the viability of a novel concept, it is crucial to investigate this causality directly [24]. The primary limitation of previous studies lies in their broad approach to manipulating DNA methylation. DNA methylation inhibitors, due to their genome-wide action, cannot conclusively establish a locus-specific elevation in promoter methylation leading directly to an increase in gene expression. However, in recent years, there has been remarkable progress in developing epigenetic editing technologies, resulting in a diverse range of tools that enable comprehensive exploration of tumour biology [25]. One particularly noteworthy advancement is the transformative impact of CRISPR-based methods on the realm of epigenetic editing. These techniques offer a revolutionary way to manipulate gene regulation precisely, creating opportunities to replicate these alterations in the context of cancer and metastasis [25], with a level of precision that was previously unattainable.

**Figure 1:**
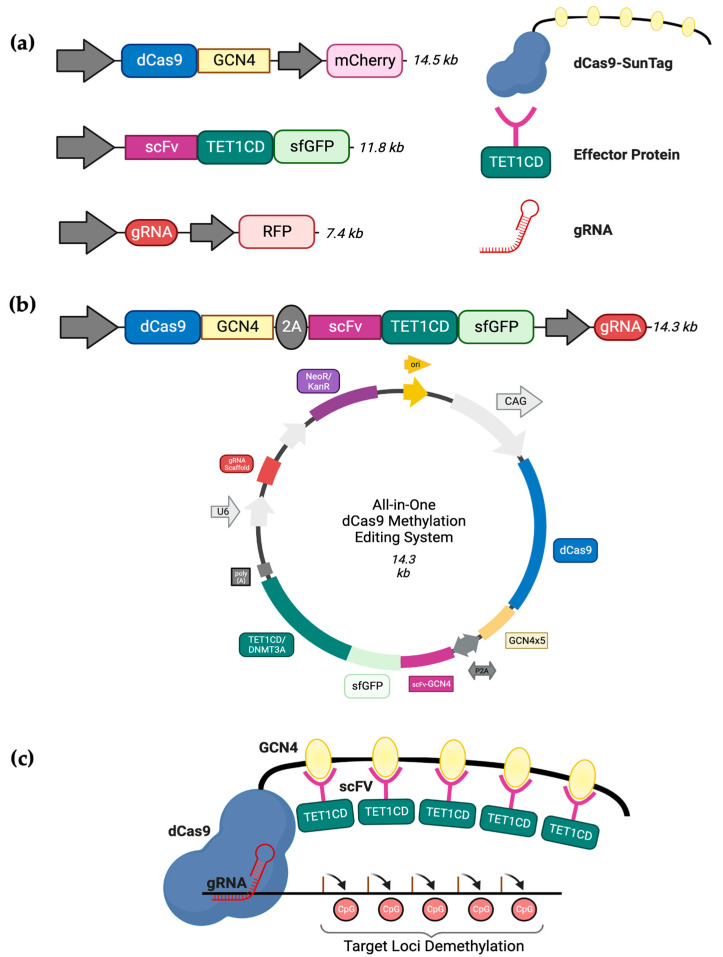
Overview of the All-in-one CRISPR-based methylation editing system. (**a**) The CRISPR-dCas9 components. Displayed here are the three main components: dCas9 construct with an associated SunTag protein scaffold that induces site-specific targeted methylation (14.5 kb); an effector protein construct (11.8 kb), either TET1CD or DNMT3A with an associated scFv domain, binds to the SunTag scaffold; and a gRNA (7.4 kb) plasmid that contains a specific target sequence. (**b**) The components of the All-in-one plasmid cloned together as a single system with the plasmid map (14.3 kb). (**c**) An example of strategy involving CRISPR-Cas9 and an amplification process based on peptide repeats to enhance demethylation. When dCas9 is linked with a peptide repeat, it can assemble numerous antibody (scFv)-linked TET1CD units, resulting in more efficient demethylation of the target due to the presence of multiple TET1CD copies.

Based on the findings from our previous work [26], attempting the concurrent transient transfection of three substantial plasmids is a challenging and time-consuming task. The primary objective of these transfection experiments was to generate enough cells for subsequent gene expression investigations and more extensive chromatin analysis. As a result, we suggest a novel approach: substituting the existing triple transfection method with a singular ‘All-in-one’ plasmid capable of expressing all three essential components of the dCas9-SunTag system. Thus, to enhance the efficiency of targeted DNA methylation editing, we obtained the All-in-one dCas9-CRISPR system described by Morita et al. [27]. This plasmid includes the dCas9-SunTag, the TET1CD-scFv effector protein, a GFP tag, and a cloning site for gRNAs, all cloned into one single plasmid (Figure 1). Similar to the three-component system [28], the dCas9 enables the RNA-guided binding of our CRISPR All-in-one methylation editing system to a specific target site without altering any DNA sequence. Additionally, the SunTag component furnishes a repetitive epitope-based framework that can secure multiple instances of our effector construct using short-chain variable fragment (scFv) domains. This interaction of the dCas9-SunTag with a particular genomic locus is guided by a distinct gRNA construct. For this project, we utilised the construct pPlatTET-gRNA2 (Addgene #82559) for demethylation and cloned our unique gRNA_472-sequence. Similarly, for targeted methylation, we replaced the *TET1* catalytic domain with *DNMT3A* using Gibson cloning, a reliable method described by Gibson et al. [29] that utilises exonucleases to assemble DNA consistently and accurately in the correct sequence. Here, we demonstrate the successful delivery and efficiency of our All-in-one dCas9 CRISPR system to induce targeted DNA methylation changes in *EBF3* and establish paradoxical gene activation. Using subsequent gene expression analysis, we provide novel clues about the function of *EBF3* in cancer cells.

The results of this study suggest that specific modifications to the DNA methylome, especially in regions that govern gene expression, could have a significant impact on modifying the gene expression patterns crucial for metastasis. This hypothesis posits that the adaptable nature of epigenetic modifications, including DNA methylation, makes it easier for tumours to acquire the specific traits needed for successful metastasis [8,10].

## 2. Materials and Methods

### 2.1. Cell Culture

The CM150-post cell line is a patient-derived line, and it was cultured in Dulbecco’s Modified Eagle Medium (DMEM) (Invitrogen, Waltham, MA, USA) with the addition of 10% foetal calf serum (FCS) and 1% penicillin-streptomycin (Gibco, Grand Island, NY, USA). WM266-4 cell line, sourced from American Type Culture Collection (ATCC. CRL-1676^TM^), was cultured in Minimum Essential Media Alpha (MEM a) (Invitrogen), supplemented with 1% penicillin-streptomycin (Gibco, NY, USA) and 10% foetal calf serum (FCS). These cell lines were grown in filter-capped cell culture flasks under standard conditions, maintained at 37 °C in a humidified atmosphere with 5% CO_2_ and 21% O_2_, as recommended. We have described the DNA methylomes of these cell lines in our previous works [26,30].

A standard protocol was employed to culture adherent human melanoma cell lines for all the cell lines used in this study. To initiate the culturing process, a frozen vial of 2 × 10^6^ cells for each cell line was thawed from liquid nitrogen storage and seeded into a 75 cm^2^ filter-cap adherent tissue culture flask (Greiner Bio One, Monroe, NC, USA) with 14 mL of the corresponding culture medium. The cells were grown until they reached over 80% confluency (i.e., 5 × 10^4^ cells/ cm^2^ before being trypsinised). The cells were then cultured in 175 cm^2^ filter-cap adherent tissue culture flasks (Greiner Bio One) with 23 mL of culture medium for experimental purposes. The time required to achieve 80% confluency in 175 cm^2^ flasks varied among cell lines and ranged from three to five days. When the cells achieved full confluence, they were prepared for transfection experiments, frozen for extended preservation, or collected for DNA/RNA extraction. The cells were monitored daily under a microscope to assess their morphology and condition and to ensure their viability at each stage of the cell culture process.

### 2.2. Plasmid DNA Isolation

In this study, we utilised the available construct pPlatTET-gRNA2 (Addgene #82559) for demethylation and cloned our respective gRNA_472-. The list of primer sequences used for inserting gRNAs and catalytic domain is provided in Table S4. Similarly, for targeted methylation, we replaced the TET1 catalytic domain with DNMT3A. This was achieved using Gibson cloning, a reliable method described by Gibson et al. [29] that utilises exonucleases to assemble DNA consistently and accurately in the correct sequence. The process was performed at a constant temperature with the aid of three enzymes: a 5′ exonuclease to create extensive overhangs, a polymerase to complete the gaps in the single-stranded regions, and a DNA ligase to join the ends of the annealed and filled gaps [29].

Achieving optimal transfection efficiency and cell viability relies heavily on extracting high-quality plasmid DNA with minimal contamination of bacterial endotoxins. The plasmid DNA for this project was isolated from DH5α *E. coli* cells. To obtain bacterial colonies for plasmid DNA isolation, we streaked DH5α glycerol stocks onto LB agar plates supplemented with 100 μg/mL ampicillin and incubated at 37 °C overnight. The next day, individual colonies were selected and cultured in 5 mL of LB broth containing 100 μg/mL ampicillin, shaken at 200 rpm, and maintained at 37 °C for 6 h. Following this, the culture was introduced into a larger volume of 400 mL, supplemented with 100 μg/mL ampicillin, and incubated overnight at 37 °C with agitation at 200 rpm. The resulting 400 mL overnight culture was then subjected to purification using the GenCatchTM Plasmid DNA Maxi Prep Kit (Epoch Life Science, Missouri City, TX, USA) in accordance with the manufacturer’s guidelines. To determine the quantity of plasmid DNA, the Nanophotometer N120 was utilised.

### 2.3. Transient Delivery of the All-in-One System

This non-liposomal mode of transient transfection was performed using FuGENE HD (Promega, Madison, WI, USA) reagent [31]. Around 5 × 10^6^ cells were initially seeded for this transfection in each 10 cm plate. On the day of transfection, 600 μL of Opti-MEM Serum Free Medium (Invitrogen) was added to a 1.5 mL tube. Then, 1 μg of the pPlatTET/DNMTA plasmid DNA was added and vortexed. Following this, 18 μL of FuGENE HD was added to the tube and immediately vortexed. The mixture was left to incubate at ambient room temperature for 10 min. Subsequently, the reagent mixture was cautiously added drop by drop to the cell plate and placed in a 37 °C incubator with 5% CO_2_ for a duration of 48 h. After the 48 h incubation period, the cell plates were examined using a microscope to observe GFP fluorescence and were then prepared for subsequent FACS analysis.

### 2.4. FACS Preparation and Sorting

The samples for FACS analysis were prepared based on previously published protocols from our lab [26,30]. Briefly, post-72 h of transfection, the cells were treated with trypsin and suspended in 250 μL of sterile auto MACS buffer containing 1 × DPBS, 1% FCS, and 2 mM EDTA. Before flow sorting, the cells were also treated with the LIVE/DEAD Fixable Near-IR Dead Cell Stain Kit. BD FACS Aria Fusion was utilised to achieve isolation of the true cell population. For each transfection, the cells were sorted based on the presence of the transfected All-in-one system, which was identified using a specific fluorescent marker (GFP in this case).

For each cell line and plasmid transfection, both negative and positive control samples were incorporated. Following that, we employed live-dead stain gating in the experiments (utilising FSC-A and APC-Cy7-A) to separate the live cell population in the negative control sample. Initially, cells were gated based on their side scatter (SSC) and forward scatter (FSC) to exclude debris, if any. The FSC measurement allows for discrimination of cells based on size. As such, the dead or apoptotic cells, which are smaller in size, can be easily eliminated. Conversely, the SSC allows for distinguishing cells based on their complexity (i.e., granularity). Following that, gating the live-dead stain used in the experiments (FSC-A and APC-Cy7-A) was utilised to gate the cells for the negative control sample and isolate the live cell population. Subsequently, the live cell population was gated based on the presence of each of the three fluorophores in their respective channels. Following this, we further isolated the true cell population based on the presence of sfGFP in the FITC channel. A total number of 27,500 and 78,190 cells were acquired using plasmids pPlatDNMT3A_gRNA_472- and pPlatTET_gRNA_472-.

FACS data analysis was conducted utilising FlowJo 10.4.2 software (FlowJo, LLC, Vancouver, BC, USA). The data, saved as .fcs files, were uploaded to the FlowJo platform. Consistent gating and compensation settings were applied to all melanoma cell lines during the analysis in FlowJo, allowing for the evaluation of cell viability and efficiency of transfection in each FACS result.

### 2.5. Assessment of Targeted DNA Methylation Editing

Elaborate methods for next-generation and specific DNA methylation analysis techniques have already been documented in our prior publications [26,32]. Consequently, this publication will not delve into further specifics regarding these methodologies.

#### 2.5.1. Sample Preparation for Illumina Mini-Sequencing

We employed the EZ DNA Methylation-Direct Kit from (Zymo Research, Orange, CA, USA) for DNA extraction and bisulfite conversion. This column-based kit employs cell lysis and bisulfite conversion, eliminating the necessity for DNA isolation, and is specifically designed for processing small cell quantities, thereby enhancing yield from a low cell input. Further, for amplicon-specific PCR, we amplified a 285 bp segment of the EBF3 promoter region using KAPA HiFi DNA Polymerase (2X Ready Mix) in a 10 μL reaction volume. The primer pairs EBF3_993_F and EBF3_993_R were utilised in a touch-down PCR approach. After PCR amplification, the presence of the product was validated by visualising it on a 2% agarose gel.

The amplified product from each sample was subsequently subjected to purification through the use of AMPure XP magnetic beads. Following the purification process, the concentration of each sample was determined using the Qubit High Sensitivity 1X dsDNA Kit (Invitrogen). Based on the quantification results, each sample was subsequently diluted to achieve a concentration of 1 ng/μL.

To enable multiplexing and demultiplexing of many samples, it is necessary to identify sequencing reads that pertain to each individual sample. A second round of PCR was therefore performed to index each sample with a unique combination of Illumina forward and reverse adaptor index sequences. Following indexing, the samples can be grouped together (multiplexed) into a unified library for sequencing. To create this library, 3 μL of each index PCR product was combined and subsequently purified again using the Qubit High Sensitivity 1X dsDNA Kit from Invitrogen. The library’s quality, including the presence of primers or non-specific products, was assessed using the Agilent BioAnalyser system and the BioAnalyser 2100 High Sensitivity DNA Assay.

#### 2.5.2. Illumina Sequencing and Data Analysis

For sequencing, a total of four samples were used. These samples included unedited and edited cells for each cell line in triplicate. Approximately 2500 cells were initially used for bisulfite conversion, which was subsequently amplified for library preparation. Mean DNA methylation levels for both the target region (nine CpG sites, 58 bp long) and the complete EBF3 amplicon (34 CpG sites, 285 bp long) were analysed. The results were also generated using three replicates for each of the samples. The relative DNA methylation change was calculated using the formula mentioned in our previous publication [26].

To analyse the raw Mini-Seq sequencing data for DNA methylation analysis, multiple bioinformatic programmes are required. The method explained in this section relies on an existing UNIX-based bioinformatic framework described previously [32]. Briefly, for each identified sample, two read files are produced, R1 and R2. These pair-ended sequencing reads are joined using PEAR (Paired-End reAd mergeR) to create full-length reads [33]. The PEAR programme uses quality scores and statistical analysis to merge reads [33]. The combined sequencing reads are subjected to a quality control check using FastQC (Babraham Bioinformatics, Babraham, UK) to identify and eliminate sequencing data with low-quality output from further analysis. To eliminate Illumina adaptor index sequences, Trim Galore! (Version 0.5.0, Babraham Bioinformatics) was employed. Each read’s alignment and methylation data were subsequently processed using the BiQ Analyser HT package [34]. The results were analysed and presented graphically with GraphPad Prism 9 (v.9.0.1). These data were also statistically analysed using an unpaired *t*-test to determine the mean difference in relation to the standard error between the two groups.

### 2.6. Sample Preparation for Gene Expression Assessment

#### 2.6.1. RNA Isolation

RNA was extracted from approximately 1000 cells following FACS sorting, utilising the RNAeasy Mini Kit from Qiagen. Initially, the cells were lysed in 350 μL of RLT lysis buffer, and then, 350 μL of 70% ethanol was added. The mixture of RLT and ethanol was thoroughly mixed before being transferred to an RNA spin column and subjected to centrifugation for 30 s at 8000× *g*. Subsequently, the eluate was discarded, and 700 μL of RW1 buffer was introduced to the RNA spin column, followed by a 15 s centrifugation at 8000× *g*. The column then underwent two additional washes, each with 500 μL of RPE buffer, at 8000× *g*. Afterwards, the column was centrifuged at maximum speed for two minutes to ensure complete membrane drying. Finally, 30 μL of RNAse-free water was applied to the column for RNA extraction, and the extracted RNA was collected in a fresh 1.5 mL Eppendorf tube by centrifugation at 8000× *g* for one minute. To determine the RNA concentration, the Qubit^TM^ RNA High Sensitivity Assay Kit (Invitrogen) was utilised with the Qubit^TM^ Fluorometer 4 (Invitrogen).

#### 2.6.2. qRT-PCR

Approximately 10 ng of RNA was converted to cDNA in a 30 μL reaction using qScript XLT cDNA Supermix Kit (Quantabio, Beverly, MA, USA) in a thermocycler. Single-stranded cDNA of 1 ng/μL was used for qRT-PCR. The SYBR Premix Ex (TaKaRa, Shiga, Japan, SYBR Premix Ex Taq) was used to prepare reactions in 20 μL volumes, and dispensed into LightCycler 480 Multiwell Plate 96-well plates. Each sample was run in triplicate as technical replicates for every experiment. The reference genes chosen for this study were RPL32 and SRP14. These reference genes were selected based on previous data from our lab group studying EBF3 expression in melanoma cells [10]. Real-time PCR reactions were run on the LightCycler 480 (Roche, Vienna, Austria). After acquiring the C_t_ values for each replicate, the values were normalised to the reference genes, RPL32 and SRP14, using the 2^−ΔΔCt^ method [35]. Subsequently, the results were analysed and presented graphically with GraphPad Prism 9 (v.9.0.1). This data was analysed using an unpaired *t*-test to determine the mean difference in relation to the standard error between the two groups.

### 2.7. RNA Sequencing Analysis

#### 2.7.1. RNA-Seq Alignment and Differential Expression Analyses

Approximately 150 ng/μL of unedited RNA and 10 ng/μL of edited RNA samples were sequenced on Illumina NextSeq 500 (AgResearch, Aotearoa, New Zealand) with 150 bp paired ends. Given the limited number of cells obtained from FACS analysis, RNA-Seq was performed without any replicates (n = 1). We have followed our previously published protocols and pipelines for RNA extraction, library preparation, and data analysis [36,37,38,39]. After sequencing, raw reads were assessed using FASTQC and MultiQC. The reads were then aligned to the human genome (GRCh37) with STAR [40]. Next, the read counting was performed using the package Subread (version 2.0.3-GCC-10.3.0 D) and function feature count. The data were subsequently analysed for differentially expressed genes (DEGs) with Edge R (version 3.40.2) [41] in R studio (version.2022.07.1+554), with a False Discovery Rate (FDR) < 0.05.

#### 2.7.2. Pathway Analyses

Differentially expressed genes common between the two melanoma cell lines were further analysed for pathway enrichment using Metascape [42]. Additionally, the ENRICHR [43] online platform was used to identify the histone and transcription factor enrichment of the common genes with several databases: Epigenomics roadmap histone modification ChIP-Seq, TRANSFAC/JASPAR transcription factor binding profiles, and ENCODE/ChEA consensus target genes. This analysis aimed to identify and elucidate the significance of enriched histone modifications and transcription factors associated with the differentially expressed common genes in our study.

### 2.8. CUT&RUN Experiments

About 2500 cells were counted for CM150-post and WM266-4 from FACS-sorted cells for each antibody (IgG, HEK27Me3 and H3K27Ac), with 500,000 cells counted for the baseline. CUT&RUN experiments were conducted following the manufacturer’s protocol with slight modifications (Epicypher, Durham, NC, USA) [44]. Eluted DNA libraries were prepared using the NEBNext UltraTM II DNA Library Prep Kit for Illumina (NEB #E7645S/L). Replicated sequencing libraries (n = 2) were sent to Genohub (Austin, TX, USA) for sequencing. A total of 35 uniquely barcoded libraries from the CUT and RUN protocol were pooled, and 150 bp paired-end sequencing was performed on the Novaseq X Plus sequencer.

## 3. Results

### 3.1. DNA Methylation at the EBF3 Promoter

**Figure 2:**
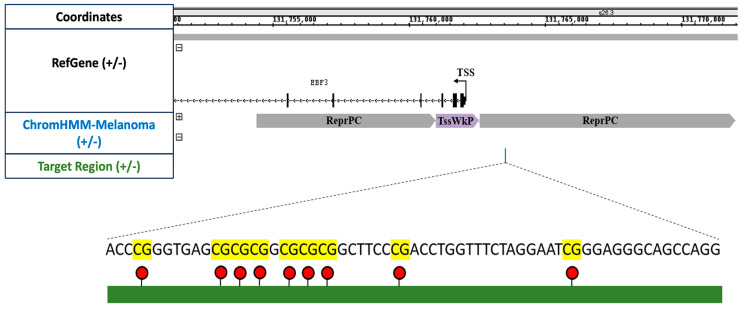
Schematic of the *EBF3* promoter and target region from the integrated genome browser. On chromosome 10q26.3, the *EBF3* promoter is depicted in the human reference sequence GRCh37/hg19. The figure also depicts the zoomed-in view of the 58 bp target region (in green), displaying the 9 CpG sites. The ChromHMM dataset indicates the promoter region to be polycomb repressed with a weak promoter at the TSS (Figure S1).

The primary consideration for efficient targeted delivery is the location of the target sequence. The precise positioning of the target sequence is essential to facilitate efficient access of the CRISPR-dCas9-SunTag All-in-one system to the target 5 mC residues and induce effective alterations in functional methylation. Previously, we extensively analysed *EBF3* methylation levels in melanoma and other cancers and identified *EBF3* as a putative epigenetic driver of cancer metastasis, particularly in melanoma [10,24]. The Integrated Genome Browser (IGB) Database illustrates the *EBF3* gene promoter in Figure 2. The position of the transcription start site (TSS) is marked at chr10:131,762,105, along with the direction of transcription. The recognised *EBF3* promoter region, which extends from chr10:131,747,593 to 131,763,801, is displayed. The target region, highlighted in green, covers chr10:131,763,530 to 131,763,587 and comprises 58 base pairs containing nine CpG sites. Moreover, in order to investigate potential changes in patterns of chromatin marks, we utilised chromatin-state discovery and gene annotation (ChromHMM) for melanoma cell lines [45]. The ChromHMM data, specific to melanoma cells, depict the predicted active promoter region (TssWkP) in purple and regions of repressive chromatin (ReprPC) in grey.

### 3.2. Targeted Locus-Specific Editing of Gene Promoter Using All-in-One System

We have utilised our previously published DNA methylation (reduced representation bisulfite sequencing, RRBS) and expression (quantitative reverse transcription polymerase chain reaction, qRT-PCR) data [7,10] to choose the suitable cell lines in this study. Consequently, cell lines CM150-post (low methylation and low gene expression) and WM266-4 (high methylation and high gene expression) were chosen on the basis of their specific methylation and expression characteristics, which were most appropriate to test the hypothesis.

**Figure 3:**
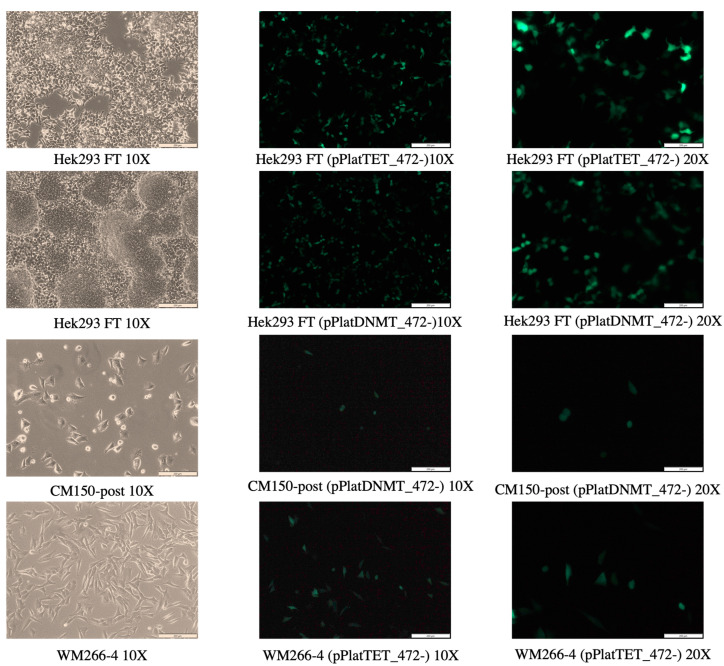
Microscopic images post-transfection. Images of transfected Hek293 FT, CM150-post, and WM266-4 cell lines using respective All-in-one construct. The images were acquired 48 h after transfection using an inverted UV microscope and the GFP filter. As seen from this figure, the cell line WM266-4 showed more GFP fluorescence (60–65%) compared to CM150-post (20–25%). Interestingly, using the same system and transfection reagent, Hek293 FT cells showed a higher GFP fluorescence (90%) when visualised under the microscope.

Transfected cells were examined under a UV microscope to detect their respective fluorescence. Using the All-in-one plasmid and FuGENE HD, GFP-positive images for the cell lines CM150-post, WM266-4, and Hek293 FT are shown in Figure 3. The cell line WM266-4 exhibited a higher GFP fluorescence (60–65%) in comparison to CM150-post (20–25%) when using the same system and transfection reagent. Interestingly, Hek293 FT cells, utilising the same system and transfection reagent, displayed notably higher GFP fluorescence (90%) compared to the melanoma cell lines when visualised under the microscope. Initial transfections with the three-component system using lipofectamine exhibited a comparatively low fluorescence signal.

### 3.3. DNA Methylation Assessment and Reproducibility of Locus-Specific Editing

**Figure 4:**
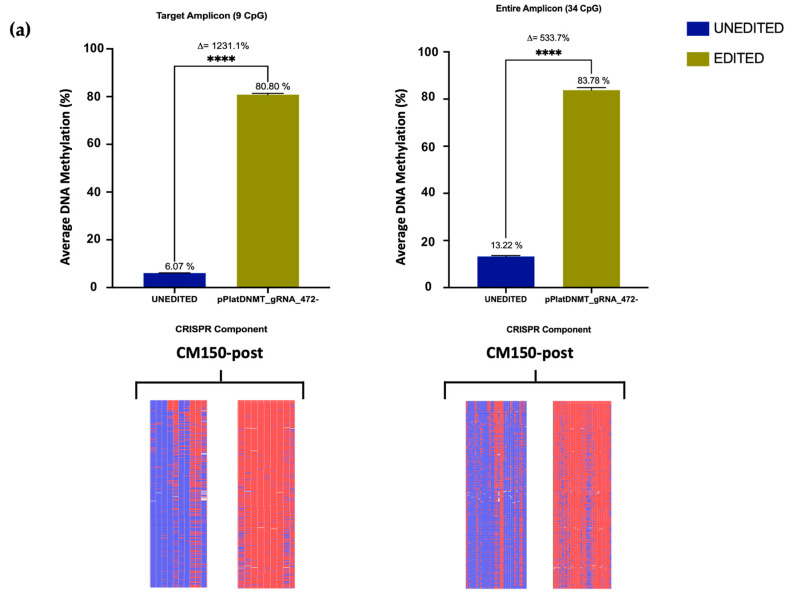

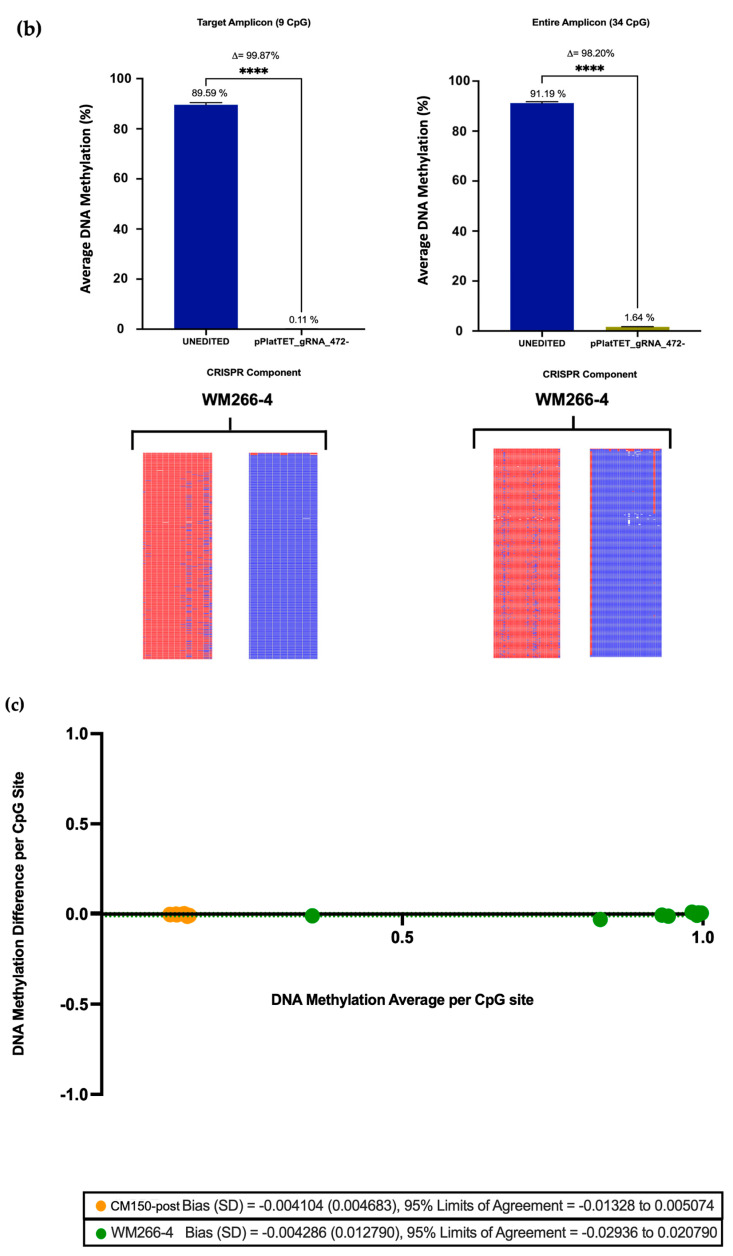
(**a**) The assessment of targeted editing using the All-in-one system in CM150-post. The graph illustrates the average DNA methylation levels for both the target region and the entire amplicon of *EBF3* in CM150-post. It compares methylation levels between unedited and edited samples, represented in absolute values. Additionally, it presents the relative change in DNA methylation levels between these two samples (denoted as ∆). Following this, heatmaps are used to depict the methylation status for each CpG site within the target region, with unmethylated sites in blue, methylated sites in red, and unaligned sites in white. These heatmaps were generated based on 500 randomly selected sequencing reads. (**b**) The assessment of targeted editing using the All-in-one system in WM266-4. Similar to CM150-post, the WM266-4 graph displays the average DNA methylation levels for target and entire amplicon region of *EBF3* with corresponding heatmaps. Additionally, the error bars in the graph indicate the mean value with its standard error (SEM) calculated from three separate biological replicates. Statistical analysis using a *t*-test yielded *p*-values > 0.05 for both cell lines (**** *p* < 0.0001). (**c**) The Bland–Altman (BA) plot illustrates a comparison between the means of CpG methylation for both unedited CM150-post and WM266-4 cell lines. This analysis involved two technical replicates for each cell line, as depicted in the figure. The proximity of each data point to the *x*-axis indicates the level of similarity between the means of the two replicates, representing the degree of difference. On the other hand, the horizontal position of each data point on the *x*-axis corresponds to the average of the two means. The two dashed lines on the plot mark the upper and lower 95% limits of agreement, which are −0.01328 to 0.005074 for CM150-post and −0.02936 to 0.020790 for WM266-4, respectively.

Employing the All-in-one methylation plasmid, we induced successful methylation of the *EBF3* target locus and the entire amplicon in the CM150-post cell line, which displays low levels of baseline DNA methylation. The absolute DNA methylation increase in the target region was from 6.07% to 80.80%, with a relative methylation increase of 1231.1%. In comparison, the absolute DNA methylation change of the entire amplicon was from 13.22% to 83.78%, with a 533.7% relative methylation increase (Figure 4a). Overall, we observed a significant change in the average DNA methylation between unedited and edited samples, thus confirming efficient editing using the All-in-one plasmid.

The All-in-one demethylation methylation plasmid was used next to edit the methylation status of WM266-4 cells, a cell line displaying high levels of baseline DNA methylation in our target region. Successful demethylation of the *EBF3* target locus and the entire amplicon was observed. Interestingly, a much more profound change in methylation status was observed with almost complete demethylation in the 58 bp target region (0.11%) and a relative methylation decrease of 99.9% in WM266-4 cells. Similarly, the DNA demethylation of the entire amplicon in the edited cells was 1.64%, with a relative methylation decrease of 98.2% (Figure 4b).

Previously, we have demonstrated effective and consistent editing of DNA methylation using a three-component system [26]. However, the aforementioned results indicated a significantly greater level of efficiency and efficacy when utilising an All-in-one plasmid to induce targeted DNA methylation alterations in the *EBF3* target region across a panel of human melanoma cell lines.

To evaluate the consistency of successive sequencing replicates for each cell line used, Bland–Altman analysis (Figure 4c) was performed. This approach compares the differences in DNA methylation (*y*-axis) relative to the mean value (on the *x*-axis) between two consecutive unedited samples, specifically focusing on each individual CpG site for every cell line. It is important to note that in this analysis, the average DNA methylation level is expressed as a proportion ranging from 0.0 to 1.0, rather than as a percentage. Based on the findings from Bland–Altman analysis (Figure 4c), DNA methylation values for each CpG site were closely clustered around the *x*-axis, suggesting minimal variation in the means of these data points. This observation was corroborated by low bias values, representing the average differences for each corresponding cell line, which were notably small, measuring at −0.0041 (CM150-post) and −0.0042 (WM266-4), respectively. Additionally, the 95% agreement limits were very narrow at −0.01328 to 0.005074 (CM150-post) and −0.02936 to 0.020790 (WM266-4). The Bland–Altman plot confirms that the inter-run variation of each data point was exceptionally low using this editing approach, exhibiting <1% variation among individual technical replicates. Moreover, no discernible patterns or alterations in variance were observed as average values increased, indicating the absence of systematic variances within the runs. In summary, the Bland–Altman assessment displays strong support for the consistent nature and reproducibility of our Mini-Seq sequencing assay across independent replicates and is suitable for methylation-status assessment.

### 3.4. Paradoxical Gene Promoter Expression Analysis

Our previous findings suggested that high *EBF3* expression is associated with high *EBF3* promoter methylation in metastatic melanoma cell lines [10]. To investigate this association and further establish the effect of targeted editing using our CRISPR system on the expression of *EBF3*, we performed qRT-PCR analysis.

**Figure 5:**
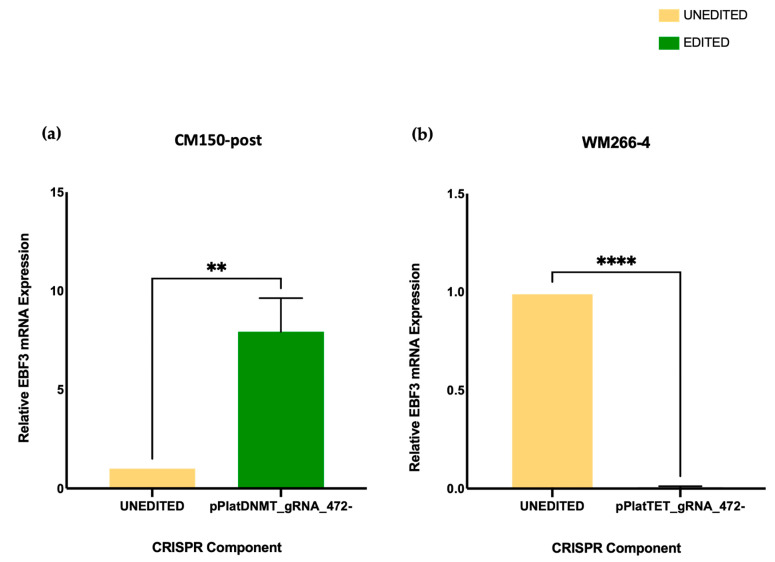
(**a**) Gene expression analysis using qRT-PCR in the CM150-post cell line. The relative *EBF3* mRNA expression displays an increase of 8-fold in expression post-editing using pPlatTET_gRNA_472-. (**b**) Gene expression analysis in the WM266-4 cell line. The relative EBF3 mRNA expression shown here displays a complete decrease in expression compared to the unedited sample. This increase and decrease in gene expression with subsequent DNA methylation, and demethylation confirms the paradoxical phenomenon of DNA methylation. Error bars represent the mean ± SD of three biological replicates. *p*-values were determined by *t*-test, *p* > 0.05 for both cell lines (** *p* < 0.01; **** *p* < 0.0001).

We determined the amplification efficiency of each primer pair by performing calculations with increasing dilutions of cDNA. Subsequently, a standard curve was generated using the *EBF3*, *RPL32*, and *SRP14* primers. The reference genes *RPL32* and *SRP14* were selected based on previous data [10] and are known to be expressed at relatively constant levels across different cell types, tissues, and experimental conditions [46]. The coefficient of determination was calculated (R2) for each primer. If *EBF3* promoter methylation can control mRNA expression, we would expect edited cells to show a subsequent change in gene expression after editing. Consistent with our prediction, the edited CM150-post (Figure 5a) cells showed an 8.0-fold increase in *EBF3* expression compared to the CM150-post unedited cells. This change in expression was consistent with the successful editing observed post-bisulfite sequencing. Similarly, the WM266-4 (Figure 5b) cell line showed almost complete transcriptional silencing, with a hundred-fold reduction in relative mRNA *EBF3* expression compared to unedited cells. Specifically, a decrease in *EBF3* methylation by editing resulted in decreased gene expression, and increased *EBF3* methylation by editing resulted in increased expression. Overall, in this context, these findings provide the first directly causal evidence for gene promoter hypermethylation inducing a paradoxical gene upregulation in gene expression.

### 3.5. Differential Gene Expression Analysis of Unedited and Edited Melanoma Cells

**Figure 6:**
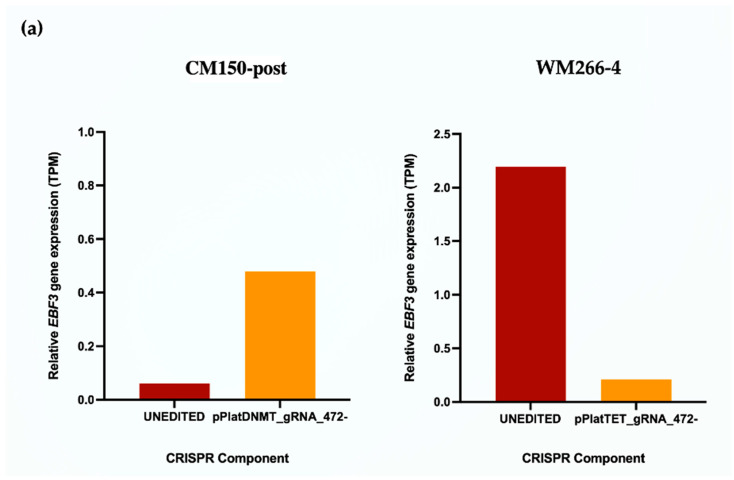

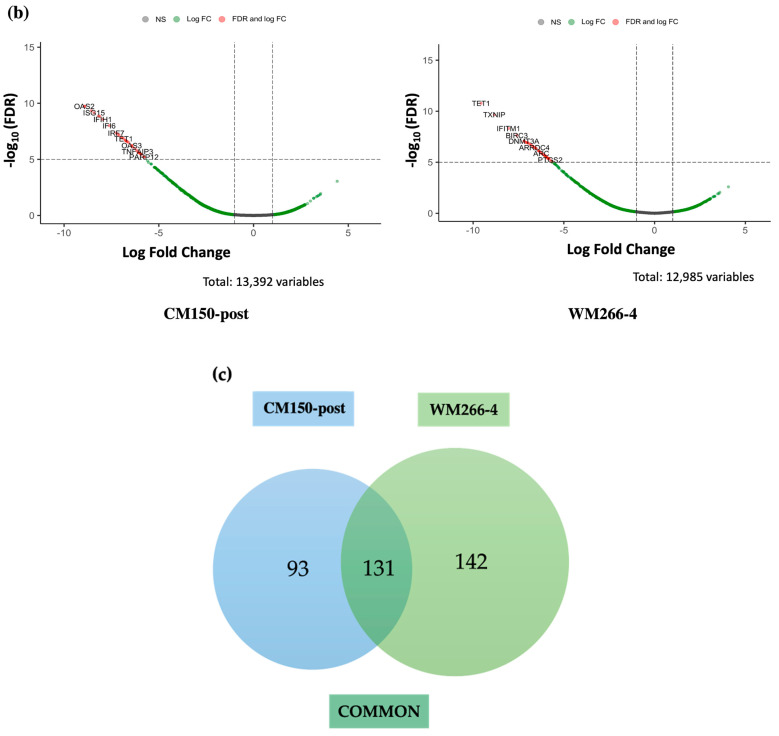

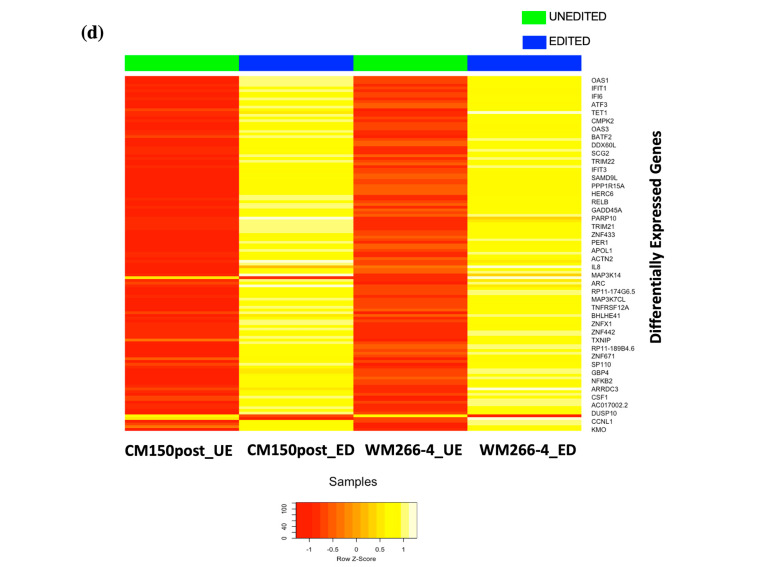
(**a**) Relative *EBF3* gene expression (TPM) displaying paradoxical changes in both melanoma cell lines. (**b**) Volcano plot showing gene expression differences between unedited and edited conditions of CM150-post and WM266-4. The *x*-axis shows the log fold change, and the *y*-axis shows the −Log_10_ FDR—corrected *p*-value. Red dots display downregulated and statistically significant DEGs with Log FC of <−1. The green dots show non-statistically significant genes but have reached the log FC threshold of <−1 or >1. Lastly, the grey dots display non-significant genes and have not reached the FC threshold, respectively. (**c**) Venn diagram of the number of common genes between the filtered DEGs of CM150-post and WM66-4. (**d**) Heatmap using row Z-score of Log_2_ (TPM +1) values of 131 DEGs for all the sample conditions in CM150-post and WM266-4 (red = downregulated, yellow = upregulated).

To analyse transcriptomic changes and validate qRT-PCR expression following targeted DNA methylation editing, we performed RNA-Seq on the same CM150-post and WM 266-4 cell lines for both unedited and edited conditions. Due to the limitation in the number of cells acquired from FACS, we sequenced a single replicate for each cell line. However, we ensured the generation of high-depth transcriptomes—the RNA-Seq generated a total of 479 million reads with an average of 119 million raw reads per sample. More than 50% of reads mapped uniquely to the human genome (GRCh37) for all samples (Table S1). First, we investigated expression changes of *EBF3* in our RNA-Seq data. The transcripts per million (TPM) method was used to calculate the relative fold change (FC) of *EBF3* in edited and unedited samples (Figure 6a). In the CM150-post cell line (Figure 6a), the edited sample showed an upregulation of 7.80 FC compared to the unedited condition, as expected. Further, the WM266-4 cell line (Figure 6a) edited sample displayed a downregulation of 10.37 FC in comparison to the unedited condition. These results further validate the paradoxical gene expression change of *EBF3* upon targeted DNA methylation editing and confirm the qRT-PCR results.

Differentially expressed genes (DEGs) were identified using the Edge R (version. 3.40.2) package [41]. In the context of Edge R for differential expression analysis, a preliminary filtration step is implemented to exclude genes with limited expression. This filtration process is typically based on the metric of counts per million (CPM), a normalisation method that scales RNA-Seq read counts by total reads. TPM, in contrast, adjusts for gene length bias, thereby normalising both sequencing depth and gene length. In our particular situation, we set a threshold of CPM > 1 for DEG analysis, indicating that genes needed to have at least 1 count per million reads in at least two samples to be included. However, *EBF3* did not meet this criterion due to its expected low expression and was excluded from the downstream DEG analysis.

We have identified the DEGs between *EBF3* edited and unedited cells for both lines. This could help uncover the broader landscape of molecular alterations and potential regulatory mechanisms associated with *EBF3* modifications in the context of the identified DEGs in CM150-post and WM266-4. A total of 224 DEGs were identified in CM150-post (FDR—corrected *p*-value < 0.05, and a mean fold change of −4.1) and 273 DEGs in WM266-4 (FDR—corrected *p*-value < 0.05, and a mean fold change of −3.7) between the unedited and edited conditions. The magnitude of fold change and statistical significance of the DEGs have been graphically represented in a volcano plot. From these 224 DEGs in CM150-post, eight genes were upregulated, whereas the remaining 216 genes were downregulated (Figure 6b). Of the 273 DEGs in WM266-4, 15 genes were upregulated, and 258 genes were downregulated (Figure 6b). Further, we have identified 131 common statistically significant DEGs between CM150-post and WM266-4 edited and unedited cells (Figure 6c). This comparison further allowed for a focused investigation into genes that consistently displayed alterations across different conditions, strengthening the likelihood of their functional significance. The heatmap (Figure 6d) reveals that, for the majority of DEGs, the unedited condition exhibited relatively low expression, whereas the edited condition displayed significantly higher expression in both cell lines.

### 3.6. Biological Pathway and Gene Ontology Enrichment Analysis of DEGs

**Figure 7:**
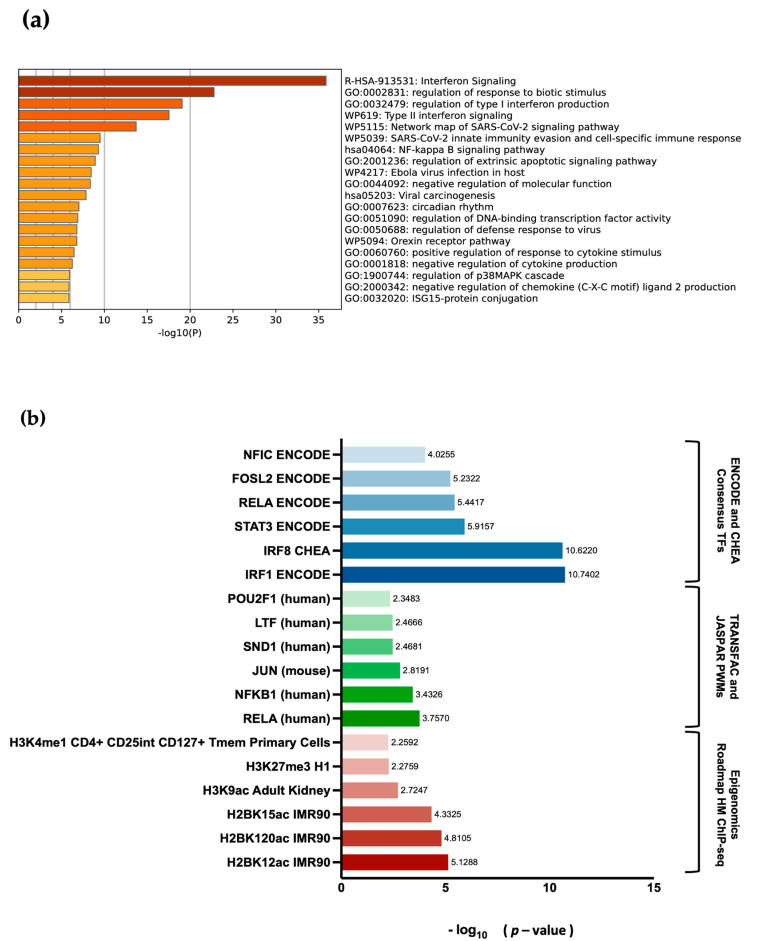
(**a**) Bar graph of enriched pathways across the common genes between the two melanoma cell lines. The pathways are analysed using *p*-values and shown here are the 20 most statistically significant enriched pathways. (**b**) Enrichment analysis of histone modifications (Epigenomics Roadmap), transcription factor binding profiles (TRANSFAC/JASPAR), and consensus target genes (ENCODE/ChEA) associated with the common genes; *p*-value < 0.05.

The list of statistically significant downregulated common genes across the CM150-post and WM266-4 melanoma cell lines was generated. Further, pathway enrichment analysis was performed using Metascape [42] to gain insights into the biological processes associated with these common genes. We aimed to identify and explore significantly enriched pathways to better understand the effect of targeted DNA methylation editing. The analysis included 131 common genes and was conducted with a significance threshold of FDR < 0.05. The Metascape analysis identified a list of 20 significantly enriched pathways for the common genes (Figure 7a and Table S2). Most of the highly significant genes (29 genes) were enriched for the interferon signalling pathway, with a −Log_10_ *p*-value of 35.85, followed by regulation of immune response (28 genes), with a −Log_10_ *p*-value of 22.79.

In addition, we conducted an analysis of histone and transcription factor enrichment using the ENRICHR tool to gain insights into the regulatory epigenetic mechanisms and potential key players in our RNA-seq dataset. We analysed the top six statistically significant enrichments (Figure 7b and Table S3). These genes were highly enriched for binding of the transcription factors in the RELA/NF-κB family, IRF1, with a −Log_10_ *p*-value of 10.74, and IRF8, with a −Log_10_ *p*-value of 10.62, both of which are highly involved in the regulation of immune responses [47]. Furthermore, these transcription factors were also enriched for H2BK12ac, an active histone mark mediated by the histone acetyltransferases (HATs), with a −Log_10_ *p*-value of 5.12.

In summary, the common genes in this analysis were strongly linked to immune responses and immune regulation, as indicated by their enrichment in interferon signaling, immune response regulation, and their association with immune-related transcription factors. Additionally, their active transcriptional state, indicated by H2BK12ac enrichment, suggests their involvement in dynamic cellular processes related to immunity.

## 4. Discussion

Previous editing of our target *EBF3* locus using a dCas9-SunTag-scFvDNMT3A/TET1CD system was able to induce notable methylation changes in three melanoma cell lines [26]. However, using the All-in-one system here, editing efficiency was significantly augmented, with an average methylation increase of approximately 80% in the CM150-post cell line (Section 3.3), rising from the initial 13.22%. Conversely, nearly complete demethylation was achieved in the WM266-4 cell line (Section 3.3), reducing the methylation level to a mere 0.11% from the initial 91.2%, respectively. A previous study utilised the dCas9-SunTag and scFv-TET1CD and indicated successful induction of targeted DNA demethylation of the *Fgf21* promoter in hepatoma both in vitro and in vivo [48]. Moreover, Josipović et al. utilised a TET1-dCas9 construct similar to ours and demonstrated precise DNA demethylation at specific target sites in ovarian cancer cell lines [49]. Taken together, the significant methylation changes observed confirm the efficacy of the CRISPR All-in-one editing system.

Recent studies, including our own, have demonstrated a paradoxical role of DNA methylation in transcriptional regulation [10,19]. In certain scenarios, promoter methylation appears to directly activate gene expression. This is supported by instances of significant gene expression levels, even when there is noticeable promoter hypermethylation. A prior study involving melanoma cell lines demonstrated a decrease in the expression of the *EBF3* gene [10] upon decitabine (DNA methylating inhibitor) treatment. However, decitabine induces global changes in methylation patterns, making it non-specific and incapable of establishing a direct cause-and-effect relationship. To offer concrete proof of gene activation driven by methylation, we opted for a precise methylation editing approach using the All-in-one system. Several early publications provide evidence for gene activation induced by hypermethylation. Early observations in the scientific literature focused on the hTERT gene, which has been widely believed to have a significant impact on telomerase activation in various cancer types [50]. Numerous studies, including those conducted on colorectal cancer, HPV-induced carcinogenesis, and brain tumours, have established a positive association between promoter hypermethylation of hTERT and enhanced transcriptional activity [50,51,52]. A recent study on prostate cancer also identified a positive corelation between hypermethylation and enhanced gene expression when evaluating the downstream regulatory impact of DNA methylation [53]. Additionally, gene body CGI hypermethylation was shown to serve as a predictive indicator for increased levels of oncogenes in hepatocellular carcinoma (HCC) mouse models [54]. Furthermore, Unoki and Nakamura [55] showed that increased methylation in the intron 1 of the tumour suppressor *EGR2*, as opposed to the promoter region, led to gene activation resembling that of an enhancer. Additionally, De Larco et al. [56] discovered a pair of hypermethylated CpG sites located upstream of the *IL8* promoter, serving as a transcriptional activator linked to the shift toward metastasis in breast carcinoma cell lines. Similarly, findings from a recent study demonstrated that intragenic hypermethylation of MMP9 is linked to the overexpression of MMP-9, thereby contributing to the development and progression of cancer [57]. Alongside these examples, our findings challenge the long-standing notion of DNA methylation as an exclusively gene-silencing mechanism. In alignment with our hypothesis, we noted enhanced *EBF3* gene expression upon promoter methylation induction in CM150-post, while this gene expression decreased upon promoter demethylation in WM266-4 cells with targeted DNA methylation editing. Whether or not this change in gene expression can subsequently alter cell phenotype is a notable aspect but was beyond the scope of this study.

RNA-seq expression analysis of *EBF3* confirmed a decrease in gene expression when demethylating the promoter region and an increase in gene expression when methylating the promoter region. These results successfully validated the paradoxical gene expression observed in qRT-PCR in the context of *EBF3.* Differential gene expression and pathway enrichment analysis revealed a strong association with immune regulatory pathways. The significant enrichment of the common genes in the interferon signalling pathway is an indication that they might play a crucial role in the body’s antiviral defense mechanisms. Interferons are signalling proteins released in response to viral infections, and they induce the expression of numerous genes that are involved in antiviral defense [58]. In the context of *EBF3* editing, our results suggests that *EBF3* may indirectly influence these genes, potentially by affecting B cell development and function, which can impact the immune response [21,59]. Similarly, the enrichment of genes related to the regulation of immune responses further underscores the importance of the common genes identified in maintaining a balanced immune system. It is likely that *EBF3* editing or dysregulation could lead to changes in the expression of these genes, impacting the body’s ability to respond to infections or regulate immune responses effectively. This is highly consistent with the fact that the common DEGs also display high enrichment for transcription factors of the RELA/NF-κB family, *IRF1* and *IRF8*, which are involved in the regulation of immune responses [47]. It is possible that *EBF3*, as a transcription factor itself, could interact or crosstalk with NF-κB family members to influence immune-related gene expression. Additionally, the enrichment of H2BK12ac indicates that these genes are actively transcribed, indicating their involvement in the ongoing and changing activities of cells related to the immune response. This could include processes such as immune cell activation, gene expression regulation, or other mechanisms crucial for the immune system’s function [60].

Our findings on the paradoxical activation of *EBF3* in melanoma, influenced by methylation changes, intersect with observations by Li et al. on the IFN pathway in cholangiocarcinoma [61]. This comparison suggests a potentially broader role for the IFN pathway in cancer, beyond its known immune functions. Further research is needed to explore how the activation of *EBF3* affects the IFN pathway in melanoma, potentially offering new insights into immune-mediated mechanisms in cancer progression and treatment strategies. However, the limited number of replicates and low expression of *EBF3* hindered our ability to accurately establish the global effect of targeted methylation editing on RNA-Seq data and its role in the enriched pathways.

Furthermore, *EBF3*, as a transcription factor, interacts with histones and chromatins to regulate gene expression and chromatin structure. As such, *EBF3* influences the accessibility of target genes and plays a role in shaping the transcriptional landscape in various cellular processes, including development, differentiation, and disease, such as cancer [62]. H3K27Me3 and H3K27Ac, two of the important histone modification marks, could be associated with the mechanism of chromatin remodelling and paradoxical gene activation. H3K27Me3 acts as a mark for the recruitment of proteins that promote chromatin compaction and transcriptional repression, leading to the silencing of gene expression alongside maintaining cellular identity [63]. Therefore, H3K27Me3 might be involved in preserving the undifferentiated state of cancer stem cells (CSCs) through the repression of differentiation-related genes like *EBF3*. This, in turn, enhances the self-renewal ability of CSCs and facilitates tumour progression [64]. In contrast, as H3K27Ac is a key mark associated with active enhancers, as such, its aberrant deposition or redistribution can result in the activation of genes that promote tumourigenesis [65]. Alterations in H3K27Ac levels and distribution are part of broader epigenetic remodelling events observed in cancer cells. These changes in H3K27Ac can occur due to dysregulation of histone acetyltransferases (HATs) and histone deacetylases (HDACs), which regulate the acetylation status of histones [65,66]. Overall, we hypothesised that editing DNA methylation at the EBF3 promoter and the associated change in gene expression could alter chromatin-bound H3K27Me3 and H3K27Ac around this region. Thus, to assess whether targeted methylation editing at the *EBF3* locus impacts the local chromatin-associated histone modifications, we performed Cleavage Under Targets and Release Using Nuclease (CUT and RUN) assays on the baseline, unedited, and edited melanoma cell lines [67]. Data visualisation using Integrated Genome Browser (IGB) (Figure S1) revealed pronounced peak enrichment within the enhancer regions that emphasised the functional relevance of H3K27Ac as a reliable indicator of enhancer activity, providing evidence that these regions are plausibly active and engaged in regulatory processes. Additionally, the robust peak enrichment observed in polycomb-repressed (ReprPC) states for H3K27Me3 baseline conditions strongly suggested that the region of interest is polycomb-repressed, as expected. Unfortunately, the absence of peak enrichment in the unedited and edited condition presented a limitation in elucidating the underlying chromatin state and interactions that could potentially contribute to a comprehension of the paradoxical gene expression phenomenon observed in *EBF3*.

In summary, the findings suggest that *EBF3*, through its putative role in B cell development and function, may have a significant impact on immune responses and immune regulation. Alterations in *EBF3* expression or activity, such as through epigenetic editing, could potentially disrupt the finely tuned immune system by influencing the expression of genes involved in interferon signalling, immune response regulation, and immune-related transcription factors. Understanding these connections could have potential implications for therapies associated with immune-related diseases such as cancer. However, further research is needed to establish the precise mechanisms and implications of *EBF3* editing on global transcriptomic regulation.

Overall, these results demonstrated the successful and efficient DNA methylation editing using an All-in-one system and also provided the first direct and causal evidence of promoter methylation causing activation of gene transcription in the context of *EBF3*.

### 4.1. Limitations and Troubleshooting

#### 4.1.1. Transfection Efficiency Improvisation

In the context of this study, we have demonstrated the methylation editing efficiency of the three-component system and the All-in-one construct. As outlined in the results presented in Section 3.2, the use of the All-in-one construct in conjunction with FuGENE HD demonstrated a substantial enhancement in transfection efficiency and the number of positively sorted cells via FACS. This improvement was particularly pronounced when compared to the utilisation of the three-component system [26] and lipofectamine 3000, confirming a significant advancement over previous methodologies. The FuGENE HD reagent is characterised by its cationic polymer properties, allowing it to create complexes with nucleic acids referred to as polyplexes or micelles when in aqueous solutions. These structures are typically less harmful to the cell membrane [68]. This unique capability to traverse the cell membrane effectively, facilitating the delivery of exogenous DNA and promoting protein expression, while simultaneously preserving the integrity and stability of the membrane, is crucial for the successful application of this reagent. Furthermore, these enhanced outcomes were accompanied by improved cell health and a reduction in cellular debris. The findings from this study were similar to a recent study that compared different transfection methods in the context of high throughput cellular assays [69]. The study revealed that FuGENE-mediated transfection was the most efficient method when compared to the effectiveness of Lipofectamine 3000 and calcium phosphate for transiently expressing two vital voltage-gated ion channels involved in pain signalling pathways [69].

#### 4.1.2. Effect of Plasmid Size and Quality in Transfection Efficiency

Our previous work using the three-component system [26] revealed another crucial insight concerning the impact of plasmid size on transfection efficiency. In this study, we found that the size of a plasmid can significantly impact transfection and editing efficiency. Plasmids that are larger in size can often face challenges in entering cells and navigating through the cellular environment, which can result in reduced transfection rates. Furthermore, within the cell, larger plasmids may be less stable, replicate less efficiently, and provide limited access to target sites for editing processes. These factors collectively contribute to lower editing efficiency when working with larger plasmids. This was seen with the limited number of cells acquired from FACS using the three-component system (33.76 kb). In contrast, plasmids with comparatively smaller sizes, such as the All-in-one construct (14.28 kb), are generally preferred as they are more efficiently taken up by cells, move more easily within the cell, and provide better access to target DNA sites for editing, leading to higher overall success rates in targeted methylation editing. As such, we employed cells acquired from transfection using the All-in-one plasmid construct for further gene expression and downstream analysis. With the All-in-one plasmids, we achieved significantly better transfection efficiency and a higher number of FACS-sorted cells. Although this cohort of cells was subsequently employed for downstream analyses, it is worth noting that the need for further amplification of cell numbers remained.

Cell toxicity during transfection can also frequently be attributed to the plasmid DNA itself [70]. The presence of residual substances such as endotoxins, salts, and lipopolysaccharides post-DNA extraction may lead to cell death and hinder the molecular outcome of lipofectamine’s cationic lipid molecules [71]. Thus, ensuring high quality and purity of DNA preparation was a significant aim of this study. The early plasmid isolation kit, Zyppy Plasmid Miniprep Kit (Zymo Research), produced low-quantity DNA leading to poor transfection efficiency. As such, we improved plasmid isolation experiments from commercially available GenCatch^TM^ Plasmid DNA columns. Using this approach, we were able to achieve a high quantity and concentration of each plasmid and generate sufficient DNA to eliminate further variations due to disparate batch preparations.

#### 4.1.3. Effect of Cell Type in Efficient Editing

In spite of the extensive application of gene transfer approaches in cellular and molecular biology research, the ability to achieve safe, efficient, and reproducible transfection of melanoma cells has remained challenging [72]. This assertion gains support from the fact that there is relatively little research conducted on the specific topic of targeted methylation editing in melanoma cell lines. This scarcity of research in the field makes it challenging to gather comprehensive insights or draw robust conclusions regarding the effects of methylation editing in melanoma cells. Furthermore, to address this knowledge gap and to understand the effect of cell type in transfection, we performed transfection in Hek293FT cells. As seen from the results (Figure 3), there was a clear increase in the sfGFP-positive cells in Hek293FT cells compared to CM150-post and WM266-4. Since melanoma cells originate from melanocytes (skin cells), they have robust defence mechanisms to protect themselves from damage, including DNA repair mechanisms and innate immune responses [73]. These defence mechanisms can act as barriers to transfection. They can rapidly degrade or eliminate foreign genetic material before it can integrate into the cell’s genome or express the desired genes [73]. Genetic heterogeneity and complex cellular properties could also contribute to poor transfection efficiency [74]. These results highlight that different cell lines may exhibit significantly varying transfection efficiencies. For instance, Choudhury et al. [75] reported up to 84.6% transfection efficiencies in HeLa cells and 56.2% in MCF7 cells using a dCas9-TET1CD single construct system, indicating substantial variability in transfection efficiency among different cell lines. However, further exploration and investigation are required to provide a more complete comprehension of the impact of cell type on transfection and editing efficiencies.

GraphPad Prism 9 (v.9.0.1). These data were also statistically analysed using an unpaired *t*-test to determine the mean difference in relation to the standard error between the two groups.

## 5. Conclusions

Here, we present a refined approach for precise DNA methylation editing in human melanoma cell lines using the All-in-one CRISPR-dCAS9 system. Furthermore, we have effectively demonstrated the paradoxical relationship between *EBF3* gene expression and DNA methylation in melanoma cell lines. Overall, this work challenges the long-standing notion that methylation is exclusively a gene-silencing mechanism and aims to establish the paradigm of paradoxical gene activation via DNA methylation for specific genes in cancer. This project also provides a platform for investigating the causative role of epigenetic drivers in cancer metastasis, both with respect to establishing optimised techniques for targeted methylation investigation and through providing insight into the role of *EBF3* in metastasis. Additionally, this study provides a prospective future strategy to develop a CRISPR-targeted methylation screen to investigate putative epigenetic drivers of metastasis in vivo. Undoubtedly, the continuous application of CRISPR-Cas9-based screening methods can offer a valuable discovery tool for identifying epigenetic regulators involved in cancer metastasis.

## Data Availability

Data are contained within the article.

## Supplementary Materials

The following supporting information can be downloaded at: https://www.mdpi.com/article/10.3390/cancers16050898/s1, Figure S1. (**a**) CUT and RUN H3K27Me3 signal and SEACR peaks in Baseline samples. The figure represents the chromatin landscape across *EBF3* gene. The promoter region spans between chr10:131,747,593-131,763,801 respectively. The *EBF3* target region is also shown in red with a black arrow. The track in dark blue indicates the H3K27Me3 signals whereas the light blue displays H3K27Me3 peaks. A comparison of profiling between two melanoma cell lines CM150-post and WM266-4 has been shown. Peaks were called for each data overlapping with ChromHMM-Melanoma dataset. (**b**) CUT and RUN for histone profiling of H3K27Ac signal and SEACR peaks in Baseline samples. The figure represents the chromatin landscape across *EBF3* gene. The promoter region spans between chr10:131,747,593-131,763,801 respectively. The *EBF3* target region is also shown in red with a black arrow. The track in orange indicates H3K27Ac signal and H3K27Ac peaks has been shown in yellow. The figure represents the chromatin landscape across *EBF3* region. A comparison of profiling between two melanoma cell lines CM150-post and WM266-4 has been shown; Table S1: Summary of RNA-seq alignments for each sample. Table S2: Top 20 pathays with their enrichment terms, related to Figure 6a. Table S3: Enrichment analysis of differentially expressed common genes between JL-PRE and WM266-4, Related to Figure 6b. Table S4: List of primer sequences used for inserting gRNAs and catalytic domains.
