# Supplementary material for: Targeted DNA Methylation Editing Using an All-in-One System Establishes Paradoxical Activation of EBF3
Source: Cancers (Basel). 2024 Feb 23;16(5):898. doi: 10.3390/cancers16050898 (PMC10930647; doi:10.3390/cancers16050898)
Supplement: Supplementary file 1 [file cancers-16-00898-s001.zip › Figure S1.pdf]

Supplementary Information

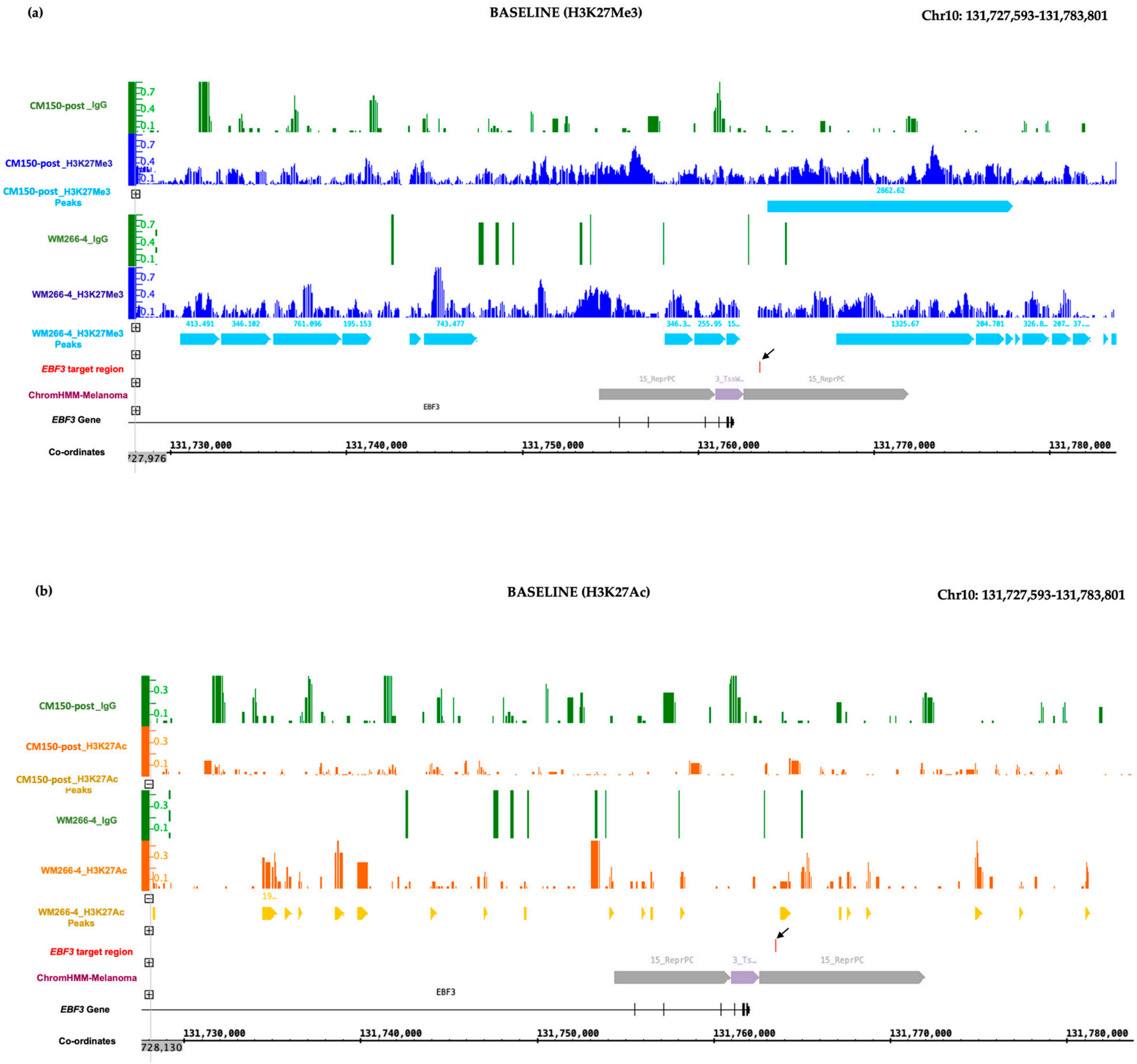

Figure S1. (a) CUT and RUN H3K27Me3 signal and SEACR peaks in Baseline samples. The figure represents the chromatin landscape across *EBF3* gene. The promoter region spans between chr10:131,747,593-131,763,801 respectively. The *EBF3* target region is also shown in red with a black arrow. The track in dark blue indicates the H3K27Me3 signals whereas the light blue displays H3K27Me3 peaks. A comparison of profiling between two melanoma cell lines CM150-post and WM266-4 has been shown. Peaks were called for each data overlapping with ChromHMM-Melanoma dataset. **(b)** CUT and RUN for histone profiling of H3K27Ac signal and SEACR peaks in Baseline samples. The figure represents the chromatin landscape across *EBF3* gene. The promoter region spans between chr10:131,747,593-131,763,801 respectively. The *EBF3* target region is also shown in red with a black arrow. The track in orange indicates H3K27Ac signal and H3K27Ac peaks has been shown in yellow. The figure represents the chromatin landscape across *EBF3* region. A comparison of profiling between two melanoma cell lines CM150-post and WM266-4 has been shown.
